# Digital staining facilitates biomedical microscopy

**DOI:** 10.3389/fbinf.2023.1243663

**Published:** 2023-07-26

**Authors:** Michael John Fanous, Nir Pillar, Aydogan Ozcan

**Affiliations:** ^1^ Electrical and Computer Engineering Department, University of California, Los Angeles, CA, United States; ^2^ Bioengineering Department, University of California, Los Angeles, CA, United States; ^3^ California NanoSystems Institute (CNSI), University of California, Los Angeles, CA, United States; ^4^ Department of Surgery, David Geffen School of Medicine, University of California, Los Angeles, CA, United States

**Keywords:** biomedical microscopy, computational imaging, computational staining, digital staining, virtual staining, quantitative phase imaging, intelligent microscopy, digital pathology

## Abstract

Traditional staining of biological specimens for microscopic imaging entails time-consuming, laborious, and costly procedures, in addition to producing inconsistent labeling and causing irreversible sample damage. In recent years, computational “virtual” staining using deep learning techniques has evolved into a robust and comprehensive application for streamlining the staining process without typical histochemical staining-related drawbacks. Such virtual staining techniques can also be combined with neural networks designed to correct various microscopy aberrations, such as out-of-focus or motion blur artifacts, and improve upon diffracted-limited resolution. Here, we highlight how such methods lead to a host of new opportunities that can significantly improve both sample preparation and imaging in biomedical microscopy.

## Introduction

Histochemical staining is an integral part of well-established pathology clinical workflows. Since thin tissue sections are mostly transparent, their features cannot be adequately observed through a standard brightfield microscope without exogenous chromatic staining. Another exogenous label commonly used to study biological specimens is formed by fluorescent probes, which enable highly specific tracking of sample components (Lichtman and Conchello, 2005) and can be used to monitor, e.g., nuclear dynamics (Kandel et al., 2020) and cellular viability (Hu et al., 2022). However, these labeling processes are time-consuming and laborious, comprising sample fixation, embedding, sectioning, and staining (Alturkistani et al., 2016). Furthermore, staining is not a perfectly repeatable procedure considering variations among human operators/technicians, and therefore the exact distribution and intensity of stains may differ from one staining operation to the next. Another disadvantage of exogenous staining is, in general, associated with their destructive nature as well as phototoxicity and photobleaching (Ounkomol et al., 2018; Jo et al., 2021; He et al., 2022), limiting imaging durations and compromising the integrity of the samples and their labels over time. Moreover, these staining procedures introduce distortions to the tissue that prevent further labelling or molecular analysis on the same regions, which presents a significant limitation in cases where multiple stains are required (Pillar and Ozcan, 2022).

An alternative approach to measuring the features of transparent biological samples is to exploit their inherent optical properties, such as autofluorescence or optical path length, in order to generate a contrast of their constituents. Autofluorescence (Monici, 2005), phase-contrast (Burch and Stock, 1942) and differential interference contrast (DIC) (Lang, 1982) microscopy offer such label-free information. Quantitative phase imaging (QPI) (Majeed et al., 2017; Park et al., 2018; Majeed et al., 2019) techniques, which provide precise phase data at the pixel level, have proven especially useful in biological applications.

Two general obstacles to using these modalities for a wider range of biomedical applications include: 1) most pathologists or medical experts have no familiarity with these kinds of images and cannot effectively interpret them, and 2) they lack the subcellular and molecular specificity that extraneous labels provide.

In recent years, largely due to the extraordinary progress in machine learning capabilities, computational staining techniques have emerged as an elegant solution to overcome these issues. Deep learning networks have been built to derive the stain of interest synthetically—whether chromatic or fluorescent—from label-free images (Rivenson et al., 2019a; Rivenson et al., 2019b; Kandel et al., 2020; Bai et al., 2022; Bai et al., 2023).

We believe the rapid expansion of such virtual staining applications and their integration with other microscopy-enhancing network models will invariably lead to transformative opportunities in biomedical imaging.

### Virtual staining

**FIGURE 1:**
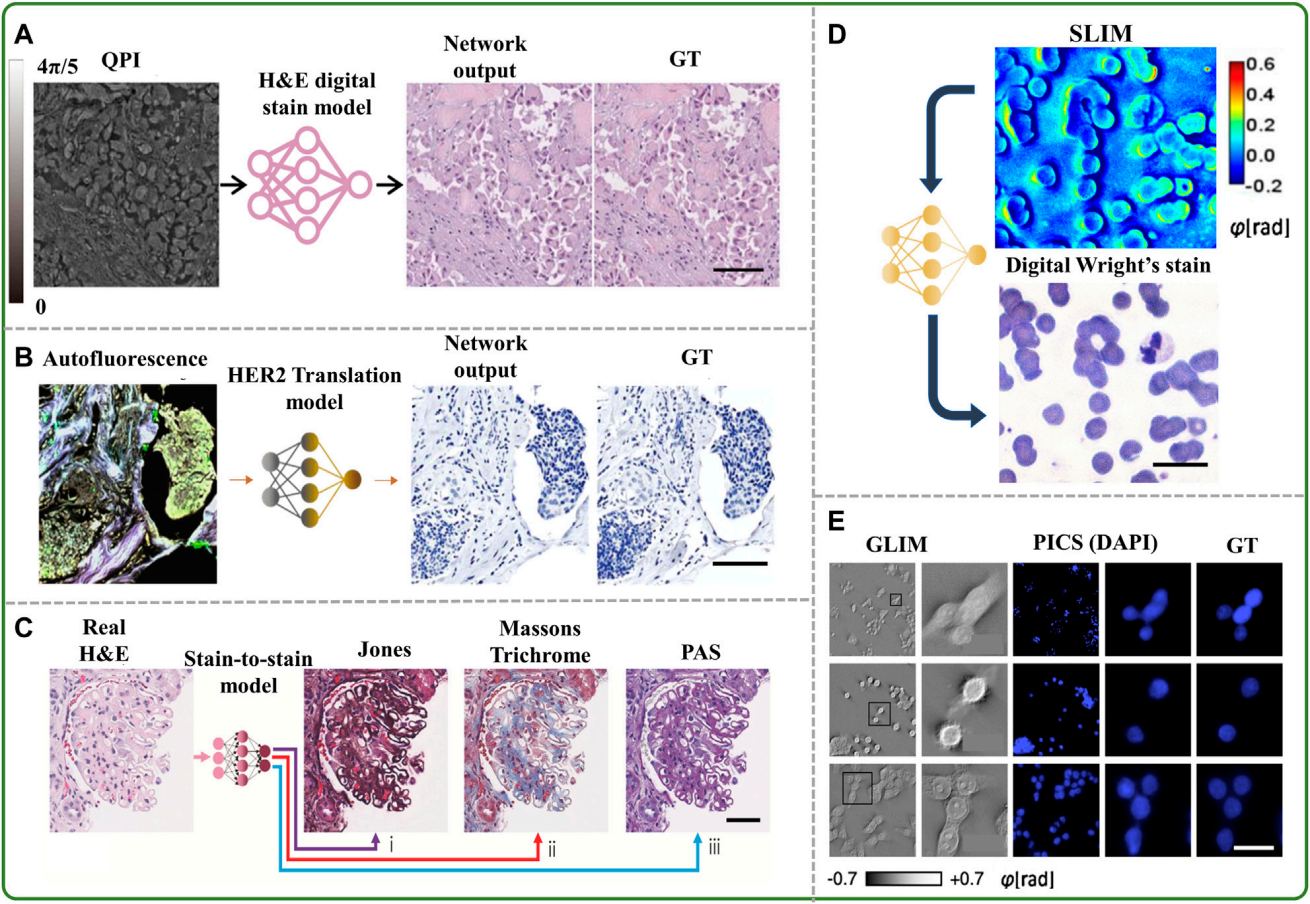
**(A)** Example of virtual staining of a QPI image to digitally generate an H&E brightfield image (Rivenson et al., 2019a), scale bar 50 μm, **(B)** example of virtual staining of an autofluorescence image to generate a HER2 brightfield image (Bai et al., 2022), scale bar 100 µm, **(C)** example of inferring special stains from an existing H&E stain (de Haan et al., 2021), scale bar 50 μm, **(D)** example of virtual staining of a blood smear quantitative phase image to digitally generate Wright’s stain (Fanous et al., 2022), scale bar 25 μm, **(E)** example of virtual staining of QPI cell images with a DAPI nuclear stain (Kandel et al., 2020), scale bar 25 µm.

Several virtual staining models have already been successfully designed and deployed, encompassing a variety of organ and staining types (Rivenson et al., 2019a; Rivenson et al., 2019b; Rivenson et al., 2020; Zhang et al., 2020; Li et al., 2021; Bai et al., 2022; Pillar and Ozcan, 2022). It has been shown that tissue biopsy images obtained with holographic microscopy or autofluorescence can be used to virtually generate the equivalents of standard histochemical stains using deep learning algorithms. In many cases, the networks involve a supervised form of the conditional generative adversarial network “GAN” (Goodfellow et al., 2020) (Figures 1A,B), which consists of a generator and a discriminator competing in a zero-sum setting. Such virtually stained slides have shown very good fidelity with their histologically stained counterparts when evaluated by pathologists (Pillar and Ozcan, 2022). This virtual staining technique greatly reduces manual labor and the costs associated with customary laboratory preparations of chemically stained tissue.

Recent advances in this emerging field also include virtual staining of label-free images obtained *in vivo* (Li et al., 2021) and stain-to-stain transformations, e.g., generating Masson’s trichome stain from the image of hematoxylin and eosin (H&E) (de Haan et al., 2021) stained tissue, as shown in Figure 1C, and even blending various stains into an intelligent amalgam for diagnostic optimization (Zhang et al., 2020).

Virtual staining has also been used on label-free images of blood smears to artificially generate the Giemsa (Kaza et al., 2022) or Wright’s stain (Fanous et al., 2022) (Figure 1D), which are commonly used to diagnose leukocyte and erythrocyte disorders. Extension of virtual staining to various fluorescent probes has also been developed to specifically detect subcellular structures of interest without the need for fluorescent tags (Ounkomol et al., 2018; Kandel et al., 2020; Jo et al., 2021; He et al., 2022; Hu et al., 2022), with deep neural networks involving mostly U-Net architectures (Kandel et al., 2020). In one such experiment, the growth of the nucleus and cytoplasm of SW480 cells was assessed over many days by applying the computed fluorescence maps back to the corresponding QPI data (Kandel et al., 2020) (Figure 1E).

Another study used virtual staining to generate semantic segmentation maps from computationally inferred fluorescence images in live, unlabeled brain cells that were subsequently utilized to decipher cellular compartments (Kandel et al., 2021). The time-lapse development of hippocampal neurons was further studied using these synthetic fluorescence signals, emphasizing the connections between cellular dry mass generation and the movements of biomolecules inside the nucleus and neurites. This technique allowed for continuous recordings of live samples without deleterious fluorescent elements.

Table 1 provides an overview of some of these virtual staining approaches, including the tested sample types and the specific advantages they offer in addition to the cost, labor, and time savings compared with traditional chemical staining methods.

**TABLE 1:** Different virtual staining methods with their applications, tested samples and specific added benefits.

Virtual staining application	Virtual staining type	Tested samples	Benefits^*^
Clinical use	Label-free to colorimetric histology stains	Salivary gland, thyroid, liver, lung, kidney Rivenson (2018), carotid Li et al. (2020); Zhang et al. (2022a), ovarian Meng et al. (2021) and skin tissue Rivenson et al. (2019a)	Uniform and repeatable staining, removal of human-induced staining artifacts, permits digital stain multiplexing on the same tissue section
	Label-free to immunohistochemical (IHC) stains	Breast Bai et al. (2022) and gastric tissue Hong et al. (2021)	Label-free biomarker for diagnostics/prognostics, tumor-stroma measurements
	Label-free *in vivo* virtual staining	Skin tissue Li et al. (2021)	Noninvasive, biopsy-free staining of skin tissue
	Label-free to cytology stains	Blood smears Fanous et al. (2022); Kaza et al. (2022) and sperm cells Nygate et al. (2020)	Simpler workflow, quantitative cell properties, less toxicity
Enhanced diagnosis	Stain-to-stain transformations	Kidney tissue de Haan et al. (2021)	Additional contrast to tissue components, highlights cells absent in deeper sections
	Digital stain blending	Kidney tissue Zhang et al. (2020)	Optimized diagnosis, digital creation of new types of stains
Research	Label-free to fluorescent stains	Colorectal cancer cells Kandel et al. (2020), HeLa Jo et al. (2021) and CHO cells He et al. (2022); Hu et al. (2022), embryonic kidney cells Ounkomol et al. (2018), and viruses Goswami et al. (2021)	Reduced photodamage, live measurements, high-throughput

*All virtual staining methods reduce cost, waste, labor, and assay time.

## Discussion

Over the past century, light microscopy has undergone a remarkable and profound transformation. It has transitioned from being predominantly descriptive and qualitative to becoming a potent tool capable of uncovering novel phenomena and elucidating intricate molecular mechanisms through a synergistic visual and quantitative approach. One key driving factor behind these advancements has been the development of numerous immunohistochemical (IHC) stains that effectively highlight specific epitopes within cells. These IHC stains have significantly enhanced diagnostic capabilities in research and clinical pathology. However, in challenging cases, several IHC stains are often employed, necessitating the use of multiple tissue slides for analysis. This becomes a bottleneck as tissue biopsies are becoming smaller in size, and there is a growing need to harness new technologies that can extract more information from limited tissue samples. With its non-destructive nature, alternative label-free optical modalities, when combined with virtual staining, hold the potential to revolutionize the histology field by enabling multiple stains from a single tissue section. This advancement opens doors for more accurate diagnosis, even when working with relatively small tissue fragments. Furthermore, a notable decrease in required reagents and chemicals, including multiple specific antibodies, can prove highly advantageous for small laboratories that lack the financial means to maintain an ever-expanding inventory of diagnostic antibodies.

The overall processing time for a typical IHC stain typically spans a couple of days. Nevertheless, certain clinical situations such as transplanted organs with suspected rejection or rapidly growing tumors necessitate a significantly expedited pathological report. As for the *virtual* staining of whole slide images (WSI), the latest cutting-edge techniques can accomplish this process within minutes. Customizing the image acquisition system and digital processing hardware could further accelerate this operation and simplify the whole measurement process. For instance, it has been shown that GAN networks can be constructed and trained to deblur out-of-focus images with high reliability in frames with axial offsets of up to +/− 5 µm from the image plane (Luo et al., 2021). To accelerate the tissue imaging process, which often consists of frequent focus adjustments during the scanning of a WSI, cascaded networks have been assembled to first restore the sharpness of defocused images that randomly appear during the slide scanning process, and then digitally perform virtual staining on these autofocused images (Zhang Y. et al., 2022). This two-step tactic enabled by a cascade of autofocusing and virtual staining neural networks is an example of how deep learning can be used to enhance not only the sample preparation and staining processes, but also the measurement, i.e., the image acquisition step.

Similarly, digital staining could potentially be coupled with the recently devised motion-blur reconstruction method named GANscan (Fanous and Popescu, 2022; Rivenson and Ozcan, 2022). This technique scans tissue slides in a continuous manner at 30-times the speed of traditional microscopy scanning, and subsequently corrects for the speed-induced motion-blur effect through a GAN-trained network. If the inputs are images generated by a label-free contrast mechanism such as QPI or autofluorescence, the results of the model could thereafter be digitally stained. This, again, could constitute a cascaded neural network architecture, first handling the deblurring operation due to rapid scanning of the tissue sample, and then virtual staining of the deblurred samples from label-free endogenous contrast to a desired virtual stain.

Another deep learning operation that can be advantageously paired with virtual staining is the enhancement of spatial resolution. It has been shown that deep learning models can be trained to convert diffraction-limited confocal microscopy images into super-resolved stimulated emission depletion (STED) microscopy equivalent images (Wang et al., 2019). To our knowledge, a concept that has not yet been realized is achieving super-resolved quantitative phase imaging through the supervised learning of fluorescent-to-phase modalities, flipping the typical direction of transformation using labeled samples. Coupling such a virtual super-resolution network with digital staining could, in principle, allow one to obtain super-resolution brightfield H&E images from ordinary label-free QPI acquisitions.

Overall, virtually transforming one imaging modality into another, along with advances in deep learning tools, has been the boon of many meaningful microscopy innovations in recent years. And there are multiple circumstances in which such a strategy of cross-modality image transformations is still unexplored or may benefit from further research.

Models may be designed to fix the various imperfections of a sample, whether optical or physical, and could thereafter be virtually stained. A consecutive GAN network would first handle artifact reconstructions/corrections, and then the stain of choice would be digitally rendered. It is also worth noting that implementing a system that enables rapid and consistent imaging, correction, and virtual staining of tissue samples would significantly enhance stain uniformity/repeatability. This is particularly crucial considering the lab-based biases present in extensive and reputable databases, such as the digital image collection of The Cancer Genome Atlas (TCGA) (Dehkharghanian et al., 2023).

The cardinal challenges to such strategies are twofold: first, a copious amount of data is required for acceptable results. Enough instances need to be included to handle the various anomalies and differences of each case; second, as this is primarily a supervised learning approach, the image pairs need to be very well registered, which might be tedious and require manual inspection and quality assurance during the training data preparation (which is a one-time effort).

## Conclusion

Virtual staining has demonstrated powerful capabilities using various modes of microscopy and will likely be implemented more and more in different bioimaging scenarios, steadily modernizing the industry altogether. The ability of virtual staining to accurately highlight tissue morphology while conserving tissue, reducing costs, and expediting turnaround time has the potential to revolutionize traditional histopathology workflows. However, for a truly disruptive virtual staining-based digitization of the well-established branches and subspecialties of pathology to occur, the technologies spanning both ends of the histological process (from sample acquisition to physician examination) need to be not only highly ergonomic, comprehensive and consistent, but also affordable and compatible with different forms of microscopy and slide scanner devices that are commercially available.

## Data Availability

The original contributions presented in the study are included in the article, further inquiries can be directed to the corresponding author.
